# Development and validation of a professionalism assessment scale for medical students

**DOI:** 10.5116/ijme.544b.7972

**Published:** 2014-11-09

**Authors:** Zalika Klemenc-Ketis, Helena Vrecko

**Affiliations:** Department of Family Medicine, Faculty of Medicine, Taborska 8, 2000 Maribor, Slovenia

**Keywords:** ethics, professional, students, validation studies

## Abstract

**Objectives:**

To develop and validate a scale for the assess-ment of professionalism in medical students based on students' perceptions of and attitudes towards professional-ism in medicine.

**Methods:**

This was a mixed methods study with under-graduate medical students. Two focus groups were carried out with 12 students, followed by a transcript analysis (grounded theory method with open coding). Then, a 3-round Delphi with 20 family medicine experts was carried out. A psychometric assessment of the scale was performed with a group of 449 students. The items of the Professional-ism Assessment Scale could be answered on a five-point Likert scale.

**Results:**

After the focus groups, the first version of the PAS consisted of 56 items and after the Delphi study, 30 items remained. The final sample for quantitative study consisted of 122 students (27.2% response rate). There were 95 (77.9%) female students in the sample. The mean age of the sample was 22.1 ± 2.1 years. After the principal component analysis, we removed 8 items and produced the final version of the PAS (22 items). The Cronbach's alpha of the scale was 0.88. Factor analysis revealed three factors: empathy and humanism, professional relationships and development and responsibility.

**Conclusions:**

The new Professionalism Assessment Scale proved to be valid and reliable. It can be used for the assessment of professionalism in undergraduate medical students.

## Introduction

Professionalism can be defined as a collection of attitudes, values, behaviours and relationships that act as the foundation of the health professional’s contract with society.^1^ It is one of the most important non-clinical topics learned and taught at all levels of medical education.  2,  3

In many curricula, at different levels of education, professionalism is a part of a hidden agenda.  4 This means that it is not present in the form of a separate subject but is often coincidentally instilled into other subjects – usually without clear learning objectives. Students therefore usually learn about it in a rather roundabout manner and rarely get assessed on what they have learned.  5

Professionalism is best taught in clinical settings (i.e. in family medicine practices, hospitals etc.) through role-modelling of teachers.  6,  7 There, students can observe the behaviour of the physicians, their interactions with patients and members of the professional team, and their actions which reflect professional norms.  8 It is known that students entering medical school already possess some attitudes towards professionalism gained from previous experience with the medical system and physicians.  9 It has already been shown that lower grade medical students are more oriented towards professionalism than those in subsequent study years, and without appropriate teaching interventions this can decline through medical school.  7 Students who interacted with consultants, colleagues and clinical staff during their clinical years were found to develop their sense of professionalism.  10

As assessment is a necessary part of education^ 11^, ^ 12^assessment of professionalism is also necessary. However, changes in professional behaviour (like changes in any type of behaviour) are difficult to assess.  13,  14 So far, many studies have tried to assess the professionalism of medical students at different levels.  7, 15- 20These studies have shown that students and trainees do have a clear insight into professionalism. Undergraduate students cited the following aspects of professionalism as important: confidentiality, good medical knowledge, practical skills,  18accountability to patients, respect for patients and their families, integrity, and prudence.  21 Similar professional features were also recognized by trainees, which emphasized accountability and respect as essential characteristics of a professional physician.  22

On the other hand, previous studies have shown that students can have different perceptions of the importance or characteristics of the elements of professionalism. In a study from the UK,^ 18^ students also put forward punctuality, hygiene and appearance as being important, although these factors are rarely cited in the literature. It is therefore important to incorporate students’ opinions about professionalism into research in this field. However, the assessment scales or questionnaires used in most previous studies were constructed on the basis of definitions of professionalism from the literature or on the opinions and attitudes of expert teachers. 

We wanted to develop and validate a scale for the assessment of professionalism in medical students, based on students’ perceptions of and attitudes towards professionalism in medicine. At the Faculty of Medicine in Maribor, students begin clinical experience in their third year. During their first year, they engage in problem-based learning during which they occasionally touch on the themes of ethics and professionalism.  23 It is known that students entering medical school already possess some attitudes towards professionalism gained from previous experience with the medical system and physicians.  9We therefore decided to engage students from the first and the fifth year, in order to detect all possible attitudes towards professionalism.

## Methods

### Study design

This study had two parts and was performed in June 2013. The first part was a qualitative study, based on focus groups and a Delphi survey. The second part was a cross-sectional observational study. 

###  Participants 

This study was carried out with medical students of the Maribor Medical School, Slovenia, with the help of Slovenian family medicine experts. The study was approved by the National Ethics Board.

### Sampling size and sampling methods

In the qualitative part, we carried out two focus groups and the Delphi survey. The first focus group consisted of five first-year medical students (three women and two men), each 19 years old. The second group consisted of seven fifth-year medical students (two men and five women), all 23 years old. Both focus groups were moderated by one of the researchers (HV) and observed by the second researcher (ZKK). 

The expert pool for the Delphi survey consisted of 20 family medicine experts, mainly teachers of family medicine and working family physicians. Ten to fifteen Delphi participants are usually sufficient if their background is homogeneous.  24

The quantitative part was designed as a cross-sectional observational study. It was carried out at the Maribor Medical School in Slovenia where all the students in each of the 6 years (N=449) who enrolled on the course for the study year 2013/2014 were contacted by email and asked to complete the questionnaire. 

### Data collection

The participants of the focus groups discussed several questions (Box 1).

Box 1: The questions discussed in focus groups

• What does the term medical professionalism mean to you?
• In your opinion, which factors are involved in medical professionalism?
• Is medical professionalism, in your opinion, part of the work of a physician, and why?
• In your opinion, how do physicians express their professionalism?
• Why is it important that physicians cherish the elements of professionalism?
• In what ways are professionalism and professional expertise associated?

A 3-round Delphi survey was conducted to validate and establish the consensus on a final professionalism assessment scale (PAS). Typically, two or more rounds of Delphi must be conducted in order to reach a valid consensus.  25 All the rounds of Delphi were presented through GoogleDoc sheets, an online survey method. The invitations were sent out by email. In the first round of Delphi, the experts were asked to mark those items which should be part of the PAS in their present formulation, those items which should not be a part of a professionalism scale, and those items that should be a part of the scale but needed editing. The researchers decided that an item should reach 90% or higher agreement to be included in the second version of the PAS. 

In the second round of Delphi, the experts were asked to mark the items on a 9-point Likert scale for clarity and necessity. For clarity, 1 point meant that the item was completely unclear and totally ambiguous and 9 points meant that the item was completely clear and totally unambiguous. For necessity, 1 point meant that the item was completely unnecessary for assessment of professionalism and 9 points meant that the item was absolutely necessary for assessment of professionalism. The researchers decided that the items’ mean scores for clarity and necessity should be 7 points or more for them to be included in the third version of the PAS. 

In the third round of Delphi, the experts were asked to provide a simple yes/no response approving the changes made in both previous rounds and the scale as a whole. Again, a 90% level of agreement was sought. 

In the quantitative part, the participants completed the questionnaires and returned them to the researcher. The questionnaires were anonymous so that the identity of the students could not be determined. The PAS’s items could be answered on a 5-point Likert scale; 1 point meant that the participant strongly disagreed with the item and 5 points meant that the participants strongly agreed with the item.

### Data analysis

The interviews derived from the two focus groups were recorded and transcribed into word documents. The transcripts were independently evaluated by both researchers (HV and ZKK). Segments of transcripts identified as important were marked as ‘verbatims’ which served as items for the development of the PAS. A grounded theory method with open coding was applied and saturation was reached.

The data from the quantitative part were analysed by the SPSS 13.0 (SPSS Inc., Chicago, IL, USA). First we performed a principal component analysis to remove any redundant or unnecessary items. The items were removed if the loadings were too weak (less than 0.39) or too general (more than 0.40 on more than one factor).  26 The item was also removed if an increase of Cronbach’s alpha of > 0.10 within the factor occurred after the deletion of the item.  27 We determined the scale’s reliability by calculating Cronbach’s alpha. 

Due to a high Cronbach’s alpha value, we calculated a composite score. We used a Baker & Hearnshaw equation  28 [(Σitems 1–22) × 100/(5 × 22)] × 1.25 – 25) to range the scale’s score from 0 to 100. In this equation, Σitems 1–22 represents the sum of the scores of 22 PAS items, 5 represents the maximum score points of each item and 22 represents the number of PAS items. The other numbers are needed for mathematical purposes in order to range the scale’s score from 0 to 100. For determining the scale’s factors, we performed a factor analysis with Equamax using the Kaiser normalization rotation method. We also calculated the Kaiser-Meer-Olkin (KMO) measure of sampling adequacy and Bartlett’s test of sphericity. For each factor, we calculated the Cronbach’s alpha. To determine the content and face validity, the Delphi method with experts (see above) was performed.

## Results

**Qualitative part**

After the focus groups and the qualitative analysis, the first version of the PAS consisted of 56 items. In the first Delphi round, 12 (600%) experts provided answers. Based on their answers, 21 out of the original 56 items were excluded, and 21 items were edited, leaving 35 items in the second version of the PAS. In the second Delphi round, 10 (50%) experts provided answers. Based on their answers, 5 out of the 35 items were excluded, so the third version of the PAS contained 30 items. In the third Delphi round, 10 (50%) experts provided answers, and based on them, no further items were excluded, leaving 30 items in the post-Delphi version of the PAS.

**Quantitative part**

The final sample consisted of 122 students from all 6 years of study (27.2% response rate). There were 95 (77.9%) female students in the sample. The mean age of the sample was 22.1 ± 2.1 years. There were 9 (7.4%) students in the first, 28 (23%) in the second, 30 (24.6%) in the third, 9 (7.4%) in the fourth, 10 (8.2%) in the fifth and 36 (29.4%) in the sixth study year. 

After the principal component analysis, we removed 8 items and came up with a final version of PAS consisting of 22 items.

The scale’s Cronbach’s alpha was 0.88 (Table 1). The mean total score was 90.9 ± 8.9 points and the median was 93.1 points. The minimum was 32 and the maximum 100 points. The KMO value was 0.846 and Bartlett’s test was highly significant (p<0.001). 

Table 1. Professionalism assessment scale: item analysis

**Table:** 

Factor	Item	Corrected item-total correlation	Cronbach’s alpha if item deleted	Factor’s Cronbach’s alpha
Factor 1 – Empathy and humanism	When managing patients the physician should put aside his/her prejudices.	0.448	0.878	0.84
	Current bad mood of the physician should not affect the management of patients.	0.479	0.879	
	Physician should have a respectful relationship towards the patients.	0.590	0.876	
	Physician should have a respectful relationship towards co-workers.	0.649	0.873	
	Physician should do his/her best to help the patient in every consultation.	0.530	0.876	
	Physician should adapt to the level of the patient's understanding.	0.425	0.879	
	Physician should be a good role model for students.	0.619	0.875	
	Each patient deserves individual management.	0.555	0.876	
	It is the physician's obligation to protect the confidentiality of the patient.	0.507	0.878	
	The physician should show interest in the patient.	0.620	0.873	
Factor 2 – Professional relationship and development	Physician should constantly engage in continuous professional education.	0.483	0.883	0.78
	Physician should set clear limits in patient communication and be able to say 'no'.	0.424	0.882	
	Physician should be able to set a clear line between private and professional life.	0.544	0.875	
	Physician should aspire to professional relationships in his/her team.	0.595	0.874	
	A lot of clinical knowledge is not sufficient to be a good physician.	0.568	0.874	
	Physician-patient communication is the basis of patient management.	0.642	0.873	
	Physician should also try to understand the patient's non-medical problems (i.e. poor financial status, family relationship problems) and include them in the consultation.	0.659	0.871	
	It is acceptable that the physician can make mistakes.	0.428	0.883	
Factor 3 – responsibility	Physician should not judge the patient by appearances.	0.585	0.874	0.60
	It is the physician's duty to present his/her professional opinion to the patient in such a way that the patient can understand and accept it.	0.427	0.880	
	The physician cannot always know what is best for each patient.	0.496	0.877	
	The physician should tell the patient frankly if there is something he/she does not know.	0.441	0.891	

Factor analysis revealed three factors: empathy and humanism (10 items), professional relationship and development (8 items) and responsibility (4 items) (Table 2) which explained 46.8% of the variance; the first factor explained 32.7%, the second 7.8% and the third 6.3%. Cronbach’s alpha of the first factor was 0.84, of the second 0.78 and of the third 0.60.

Table 2. Factor analysis of professionalism assessment scale

**Table:** 

Item	Factor 1 – empathy and humanism	Factor 2 – professional relationship and development	Factor 3 – responsibility
When managing patients the physician should put aside his/her prejudices.	0.511	0.217	0.101
Current bad mood of the physician should not affect the management of patients.	0.493	0.090	0.370
Physician should have a respectful relationship towards the patients.	0.818	0.103	0.130
Physician should have a respectful relationship towards co-workers.	0.702	0.285	0.187
Physician should do his/her best to help the patient in every consultation.	0.486	0.122	0.417
Physician should adapt to the level of the patient's understanding.	0.368	0.258	0.187
Physician should be a good role model for students.	0.652	0.411	0.025
Each patient deserves individual management.	0.633	0.041	0.369
It is the physician's obligation to protect the confidentiality of the patient.	0.592	0.071	0.299
The physician should show interest in the patient.	0.551	0.211	0.407
Physician should constantly engage in continuous professional education.	0.051	0.415	0.155
Physician should set clear limits in patient communication and be able to say 'no'.	0.045	0.686	-0.063
Physician should be able to set a clear line between private and professional life.	0.287	0.556	0.204
Physician should aspire to professional relationships in his/her team.	0.470	0.593	0.011
A lot of clinical knowledge is not sufficient to be a good physician.	0.439	0.472	0.147
Physician-patient communication is the basis of patient management.	0.493	0.481	0.223
Physician should also try to understand the patient's non-medical problems (i.e. poor financial status, family relationship problems) and include them in the consultation.	0.296	0.504	0.453
It is acceptable that the physician can make mistakes.	-0.169	0.629	0.311
Physician should not judge the patient by appearances.	0.363	0.247	0.527
It is the physician's duty to present his/her professional opinion to the patient in such a way that the patient can understand and accept it.	0.197	-0.012	0.724
The physician cannot always know what is best for each patient.	0.236	0.299	0.473
The physician should tell the patient frankly if there is something he/she does not know.	-0.120	0.118	0.620
Eigenvalue	7.463	1.510	1.316
Variance (%)	32.720	7.781	6.268

Several items were perceived to be of the highest importance: a current bad mood of the physician should not affect the management of patients; the physician should have a respectful relationship towards the patients; it is the physician's duty to present his/her professional opinion to the patient in such a way that the patient can understand and accept it; and it is the physician's obligation to protect the confidentiality of the patient. Two items were perceived to be the least important: the physician cannot always know what is best for each patient, and the physician should tell the patient frankly if there is something he/she does not know (Table 3).

Table 3. Respondents’ evaluation of professionalism assessment scale

**Table:** 

Item	Mean score ± standard deviation	Median (min, max)	Skewness	Kurtosis
When managing patients the physician should put aside his/her prejudices.	4.7 ± 0.6	5.0 (2,5)	-2.391	7.687
Current bad mood of the physician should not affect the management of patients.	4.9 ± 0.4	5.0 (3,5)	-2.728	7.086
Physician should have a respectful relationship towards the patients.	4.9 ± 0.4	5.0 (1,5)	-8.892	86.616
Physician should have a respectful relationship towards co-workers.	4.8 ± 0.6	5.0 (1,5)	-3.229	13.777
Physician should constantly engage in continuous professional education.	4.6 ± 0.7	5.0 (1,5)	-2.467	7.790
Physician should do his/her best to help the patient in every consultation.	4.8 ± 0.5	5.0 (2,5)	-2.610	7.682
Physician should not judge the patient by appearances.	4.6 ± 0.7	5.0 (2,5)	-2.092	3.999
Physician should adapt to the level of patient's understanding.	4.5 ± 0.8	5.0 (1,5)	-1.802	3.928
Physician should set clear limits in patient communication and be able to say 'no'.	4.6 ± 0.7	5.0 (2,5)	-1.942	3.916
Physician should be a good role model for students.	4.8 ± 0.5	5.0 (2,5)	-2.950	10.293
Physician should be able to set a clear line between private and professional life.	4.5 ± 0.7	5.0 (2,5)	-1.570	2.236
Physician should aspire to professional relationships in his/her team.	4.7 ± 0.6	5.0 (2,5)	-1.902	3.071
A lot of clinical knowledge is not sufficient to be a good physician.	4.6 ± 0.8	5.0 (1,5)	-2.292	5.505
Physician-patient communication is the basis of patient management.	4.7 ± 0.6	5.0 (2,5)	-1.892	3.604
Physician should also try to understand the patient's non-medical problems (i.e. poor financial status, family relationship problems) and include them in the consultation.	4.5 ± 0.8	5.0 (1,5)	-1.469	2.303
Each patient deserves individual management.	4.8 ± 0.5	5.0 (2,5)	-3.242	13.114
It is the physician's duty to present his/her professional opinion to the patient in such a way that the patient can understand and accept it.	4.9 ± 0.4	5.0 (3,5)	-2.862	7.968
The physician cannot always know what is best for each patient.	4.1 ± 0.9	4.0 (1,5)	-0.772	0.349
It is the physician's obligation to protect the confidentiality of the patient.	4.9 ± 0.4	5.0 (3,5)	-3.054	9.330
The physician should show interest in the patient.	4.6 ± 0.7	5.0 (1,5)	-2.846	10.656
The physician should tell the patient frankly if there is something he/she does not know.	3.9 ± 1.1	4.0 (1,5)	-0.747	-0.286
It is acceptable that the physician can make mistakes.	4.6 ± 0.8	5.0 (1,5)	-2.150	4.117

##  Discussion

**Summary of main findings**

This study proved the new PAS to be reliable and valid when assessing professionalism attitudes in undergraduate medical students. Three factors – empathy/humanism, professional relationship/development, and responsibility – emerged as the key factors of the scale. It seems that attitudes of undergraduate students towards professionalism are associated with gender and age.

Comparison to other tools

Our tool for the assessment of professionalism proved to have good internal reliability. Also, it has good construct validity as the corrected item correlation was above 0.40 for each item.

This indicates that the PAS can be used as a whole and its total score can be calculated when detecting possible associations or correlations with different variables. The internal reliability of other scales for the assessment of professionalism in other studies ranged from 0.71 to 0.86.  15,  16, 29, 30 Our tool seems to cover several professional themes which loaded to three factors: empathy/humanism, professional relationship/development, and responsibility. A USA charter of professionalism, published in 2002, described three fundamental principles of professionalism: patient welfare (altruism, trust, patient interest), patient autonomy (honesty, patient empowerment) and social justice.  31 In addition to these principles, our tool also recognised the need and self-awareness of medical students for continuous professional education. Factor analysis of other tools revealed a different number of factors: from three29 to as many as eight.  21 It seems that the number of factors does not affect the comprehensiveness of the tools, as even though some of the tools have a smaller number of factors, they still cover most professionalism features.  29, 30

The students scored high on the PAS, and it therefore seems that they are aware of medical professionalism. The most highly scored items came from the empathy/humanism domain. This is in line with the work of Al-Eraky et al^ 16^ and Tsai et al.^ 21^ On the other hand, the lowest scored items came from the domain of responsibility, which is not in line with Al-Eraky et al.  16 However, El-Eraky et al^ 16^ studied medical students and interns, which might have accounted for this difference. At the undergraduate level, the students may not yet be fully aware of their responsibilities as physicians.

**Factor 1**

The first factor covered empathy and humanism and explained most of the variance. Its internal reliability was good. Empathy and humanism factors have also been recognised in other tools as upholding principles of integty,  15 respect and relationships with others,  15 honour/integrity,  16, 32 equity,  32 commitment to care,  21 patient-oriented issues,  21 and respect for others.  21Empathy and humanism items are also one of the fundamental principles of medical professionalism. 31 It seems that empathy and humanism items can be found in most professionalism assessment scales, but in different factors; nevertheless, this implies that these professionalism features are fundamental and are always recognised by students, and so should be a part of every professionalism assessment scale.

**Factor 2**

The second factor covered professional relationships and development. Its internal reliability was good. These items were also recognised in other tools as relationship with others,  15 duty/accountability,  16, 32 excellence/autonomy,  16 duty,  32 habit of professional practice^ 21^ and respect for others.  21 One could anticipate that undergraduate students might not be fully aware of the need for professional development as this is usually a feature of long-life learning.  33 However, according to the results of our and similar studies,  16,  21 this is not the case. 

**Factor 3**

The third factor covered responsibility. Its internal reliability was acceptable. Responsibility was recognised also in other tools as responsibility,  15 accountability,^ 15^altruism,  16,  32 righteous^ 21^ and rule-abiding.  21Responsibility is one of the fundamental features of a professional physician. This was also recognized by the students in the qualitative part of the study, even though later on these items were perceived as the least important. 

**Implications of the study**

Our study indicates that a valid and reliable scale can be developed from the analysis of students’ attitudes towards medical professionalism. Factor analysis showed that the PAS covers the most essential features of medical professionalism. When dealing with scales for the assessment of professionalism, we have to bear in mind that no single tool can include all traits and behaviours of medical professionalism, as this is a complex construct^ 16^ and can also be perceived differently by different populations. 

The PAS has been designed and tested in a sample of undergraduate medical students. As it has been derived from the qualitative analysis of the interviews with this population, we can claim that it can be successfully used with undergraduate medical students. It can be used to assess the students’ attitudes towards professionalism at the beginning of the study and in formative assessment. It can also be used for students’ self-assessment. Further studies are needed to determine its use in medical education and also in other populations, such as residents, practising physicians, and also in patients, nurses and other healthcare professionals. 

The bivariate analysis showed some associations with gender and age/study year. Female students, older students and those in later study years scored higher. The latter was expected, as medical education should constantly work on building a professional physician. However, further studies are needed to confirm such associations. 

**Limitations of the study**

The sample size was sufficient for the validation study; usually, a sample size should consist of at least 80 participants.  34 However, some authors^ 35^ argue that an adequate sample should consist of at least 300 participants. In our study, the reliability and validity of the PAS indicates that the sample size was sufficient. Empathy was revealed as the main factor on the scale; due to the fact that most of the sample was female, this finding could be a consequence of a gender bias, so this result should be interpreted with care. Also, the response rate in quantitative part of the study was low, which could have affected the external validity of the study due to an auto-selection bias. 

According to skewness and kurtosis, the distribution of the data was probably not normal, which further confirms that the sample could be auto-selected.

We carried out several rotations in factor analysis in order to get the best solution. In the end we chose the Equamax rotation which is also used when dealing with orthogonal items. Some items of the scale showed share loadings on at least two factors. We decided on where they should be placed according to the content of the factor itself. However, it is also true that professionalism items often overlap, as professionalism features are associated with each other and cannot be regarded as separate entities. 

##  Conclusion

The new professionalism assessment scale proved to be valid and reliable. It can be used for the assessment of professionalism in undergraduate medical students in terms of summative and formative assessment and for self-assessment. Further studies are needed to determine its use in undergraduate settings and to study the associations of students’ attitudes towards professionalism and the undergraduates’ characteristics.
